# Speckle patterns formed by broadband terahertz radiation and their applications for ghost imaging

**DOI:** 10.1038/s41598-021-99508-1

**Published:** 2021-10-08

**Authors:** Lev Leibov, Azat Ismagilov, Victor Zalipaev, Boris Nasedkin, Yaroslav Grachev, Nikolay Petrov, Anton Tcypkin

**Affiliations:** 1 Laboratory of Quantum Processes and Measurements, ITMO University, St. Petersburg, Russia 197101; 2 Laboratory of Digital and Display Holography, ITMO University, St. Petersburg, Russia 197101

**Keywords:** Imaging and sensing, Terahertz optics, Optics and photonics

## Abstract

Speckle patterns can be very promising for many applications due to their unique properties. This paper presents the possibility of numerically and experimentally formation of speckle patterns using broadband THz radiation. Strong dependence of the statistical parameters of speckles, such as size and sharpness on the parameters of the diffuser are demonstrated: the correlation length and the mean square deviation of the phase surface inhomogeneity. As the surface correlation length is increasing, the speckle size also increases and its sharpness goes down. Alternatively, the magnification of the standard deviation of the surface height leads to the speckle size diminishing and growth of the speckle sharpness. The dimensions of the experimentally formed speckles correspond to the results of numerical simulation. The possibility of utilizing formed speckle patterns for the implementation of the ghost imaging technique has been demonstrated by methods of numerical modeling.

## Introduction

Speckle pattern formation is a phenomenon that occurs in situations where a coherent light reflects or transmits through a rough surface or a turbid medium^1,2^. Despite the fact that the occurrence of speckle-noise can affect negatively for some imaging techniques (e. g. optical coherence tomography and LIDAR technologies), there are fields in which usage of speckle patterns can be very promising: it applied for image retrieving in speckle metrology^3^, single pixel imaging^4^, for ghost imaging^5–8^, cryptography technique^9^ etc. In turn, terahertz (THz) radiation, due to the optical properties of various materials in this frequency range, as well as the features of individual properties of the radiation itself, is becoming more popular in quality control systems^10–12^, safety^13–16^ and biomedicine application^17–19^, communication^20–23^.

The formation of speckle patterns for THz radiation begins to be studied in detail only at the present time. The first speckle structures in the THz region were first obtained using monochromatic continuous THz radiation and discussed in papers^24,25^. All these results were experimentally tested using a free electron laser (FEL). In^25–27^ formation of speckle patterns by reflection FEL THz radiation from a random rough surface was also considered. The use of other sources of monochromatic radiation is presented in^28^. In this paper, for the formation of speckles, metal disk with a set of various random amplitude images around the disk circumference was used. In works^25–28^, the possibility of application such speckle structures for the ghost imaging technique was demonstrated. High energies, various amplitude and phase objects transmission are the main advantages of monochromatic continuous THz radiation.

Usually, pulsed THz radiation has low energies^29,30^, but there are already widespread methods of generating high-energy radiation, for example, such as in organic crystals^31,32^ or by filamentation in gases or liquids^33,34^. The generation of speckle structures by pulsed THz radiation has not been studied yet. However, it should be noted that mask formation methods using nonlinear transformations in the crystal for generating the pulsed THz radiation exist and could be found in^35^. In this case, spatial light modulator, which modulate pump radiation is used. The modified beam is directed to the crystal for generating the THz field. This allows the ghost imaging technique in the THz range realization. A variant of amplitude modulation of probe radiation was proposed as a separate method for implementing THz imaging with sub-wavelength accuracy^36^. Producing of speckle patters for terahertz spectral range is of great interest to various applications related to terahertz imaging. This aspect motivated us to study speckle-pattern formation by broadband pulsed THz radiation.

In this work we present a numerical model describing the formation of speckle patterns using broadband pulsed radiation of THz spectral range. Some of statistical characteristics of obtained speckle patterns, such as size and sharpness, and their dependencies on parameters of random phase screen are discussed. Some limitations were shown that affect the size of speckle patterns formed by broadband THz radiation. Experimental verification of the formation of speckle patterns was carried out. Furthermore, in the paper the possibility of utilizing THz speckle patterns in pseudothermal light ghost imaging algorithm was numerically demonstrated. An opportunity of application of numerically formed speckle patterns for ghost image reconstruction even in case of working with broadband THz pulses is also shown. Current mathematical model can be used not only for radiation of the THz spectral range, but for any wavelength bands in wave optics and in radio physics as well. Moreover, this model is capable of dealing with the broadband pulsed radiation. Thus, in contrast to standard optical bands, it is possible to work with THz pulses with a significant spectral width. In this work, we use pulses with a spectral width of the order of 1 THz (from 0.2 to 1.2 THz). It is also worth noting that in case of ghost imaging it is important to take into account the relationship between the characteristic values of the wavelengths in the selected pulse spectrum and the dimensions of the object. This relationship is fundamental for ghost imaging, and the quality of the reconstructed image depends on the choice of the radiation wavelength used to reconstruct the image of a particular object. The dimensions of the spatial inhomogeneities of the phase plate are also related to the choice of the object dimensions and the radiation wavelength.

## Mathematical model

**Figure 1:**
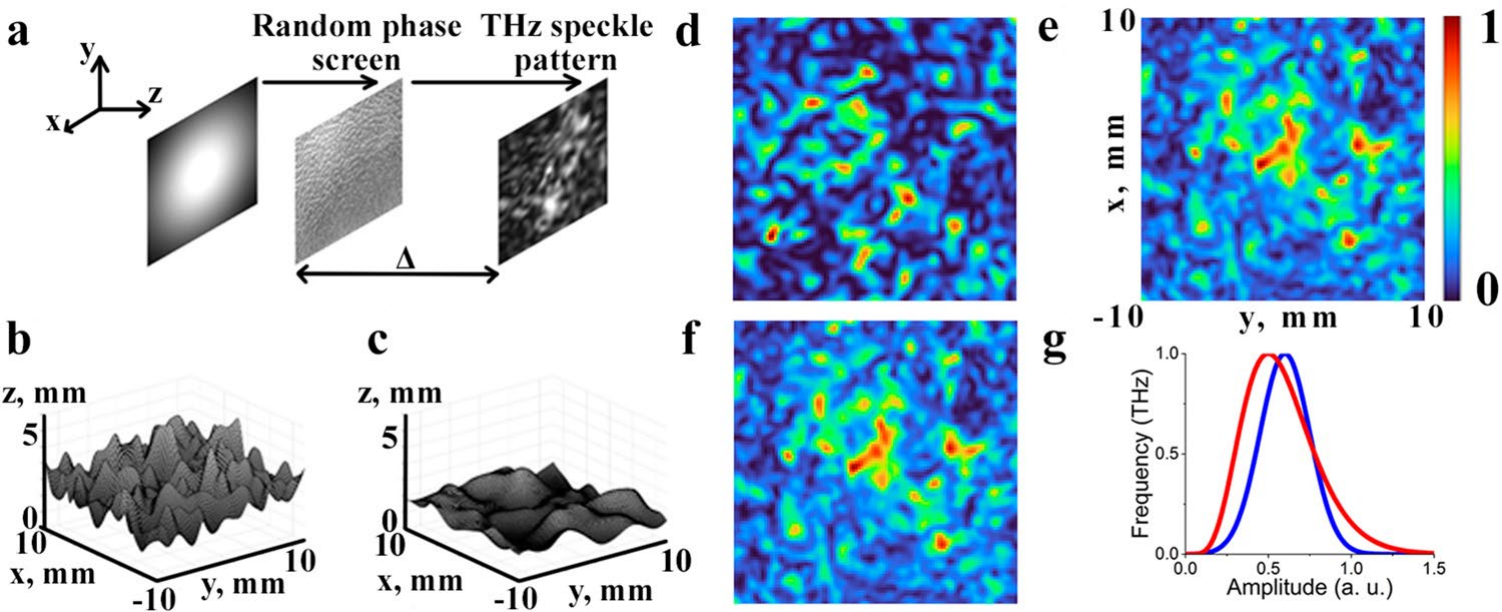
(**a**) Scheme of acquisition of speckle patterns for monochromatic THz radiation: beam with Gaussian intensity distribution transmits through the random phase screen and propagates over the distance $\Delta =5$ cm. (**b**,**c**) Two samples of Gaussian random rough surface. Surface has large value of ${h_{RMS}}$ and low value of *Cl* (**b**), low value of $h_{RMS}$ and large value of *Cl* (**c**). Speckle patterns produced by (**d**) monochromatic THz radiation with frequency $\nu =0.7$ THz, produced by pulsed THz radiation with symmetric spectrum (**e**) and spectrum with shifted central frequency (**f**). Spectrum (**g**) with a blue curve has a symmetric Gaussian shape, spectrum with red curve has gaussian shape with shifted central frequency. As a material of plate with random rough surface on its back side with refraction index ${n}=1.55$ for THz spectral range was used, parameters of the surface are ${h_{RMS}}=0.3$ mm and ${Cl}=0.9$ mm. The distance between random phase screen and speckle pattern observation plane is $\Delta =5$ cm

In this work we study formation of speckle patterns using broadband pulsed THz radiation. There are a lot of approaches for obtaining of random light fields^37–39^. One of the most widespread methods propose using random refractive inhomogeneity for light modulation^40^. In our paper we expand this approach for broadband THz radiation and we form speckle patterns by means of transmission of THz pulses through the transparent plate with random rough surface on its back side. This random phase screen provides a phase modulation of THz pulses, which leads to the appearance of speckle patterns at some distance behind the screen, as shown in the Fig. 1a. In this section, a numerical model of speckle formation is presented and the dependence of the speckle parameters on the characteristics of the transparent plate with random rough surface is investigated.

### Speckle pattern formation in the monochromatic and pulsed THz radiation

In the first part of the mathematical modeling, the formation of speckle structures by THz radiation, both monochromatic and pulsed, is considered. To describe speckle pattern formation for the monochromatic THz radiation we use model of Gaussian beam with the field distribution $E_{in}$ which can be described by:

1 $$\begin{aligned} E_{in}(x,y,z)=\exp (ikz)E_{0}\frac{-ika^{2}}{z-ika^{2}}\exp \left( \frac{ik(x^{2}+y^{2})}{z-ika^{2}}\right) , \end{aligned}$$

where *x*, *y*, *z* are the spatial coordinates, *a* is the spatial parameter of Gaussian beam related to the beam waist, $E_{0}$ is the initial amplitude of beam, *k* is the wave number.

Suppose, that a Gaussian beam that is incident to the random phase screen at $z=0$. Using the phase shift introduced by random phase screen, the field distribution of beam right after the random phase screen is evaluated in the following way^41^:

2 $$\begin{aligned} E (x,y,z=0)= E_{in}(x,y,z=0) \cdot \exp \left( i\frac{2\pi }{\lambda }(n-1)h(x,y)\right) , \end{aligned}$$

where ${\lambda }$ is a wavelength, *n* is the refraction index of material of plate with random rough surface on its back side, $h(x,\,y)$ is the function of height profile of random rough surface. Gaussian random rough surface model is used for formation of rough structure^42^. This surface has two parameters which describe the height profile. The first parameter is the root mean square deviation of the surface height $h_{RMS}$, and the second parameter is the horizontal correlation length *Cl* of the surface, which shows the frequency of spatial inhomogeneities of the surface. Formation of Gaussian random rough surface $h(x,\,y)$ is described by the following equation:

3 $$\begin{aligned} h(x,y)=\frac{2L}{\sqrt{\pi }NCl}F^{-1}\left[ F(H_{1})\cdot F(g)) \right] , \end{aligned}$$

where *L* is the length of the surface side, *N* is a size of the matrix $h(x_{i},\,y_{j})$ ($i,j=1,2,\ldots ,N$) obtained due to discretization, $H_1$ is $N\times N$ matrix of random numbers with Gaussian distribution $\varphi$ and standard deviation ${h_{RMS}}$: $H_{1}=h_{RMS}\cdot \varphi$, *g* denotes a Gaussian filter $g(x,y)=\exp (-2(x^{2}+y^{2})/Cl^{2})$ with correlation length *Cl*; *F* and $F^{-1}$ are direct and inverse Fourier transforms, respectively. Figure 1b,c demonstrates two examples of Gaussian random rough surface *h*(*x*, *y*) with different values of $h_{RMS}$ and *Cl*.

While propagating along the *z* axis over the distance $\Delta$, the intensity profile of the modulated beam becomes random, which results in a speckle pattern formation at the observation plane $z=\Delta$. Propagation of the modulated beam along *z* axis was calculated by means of the well-known two methods: Fresnel propagation formula (see “Methods” section) and the angular spectrum approach (plane wave decomposition, see “Methods” section). It is worth remarking that both methods provided the same numerical results for speckle pattern formation.

Consider formation of speckle patterns for pulsed THz radiation. Taking into account all spectral components of THz pulse with spectrum $S(\nu )$, we have:

4 $$\begin{aligned} E(x,y,\Delta ,t)=\frac{1}{2\pi }\int \limits _{-\infty }^{\infty }{\exp (-i2\pi \nu t)S(\nu )E(x,y,\Delta ,2\pi \nu /c)}d\nu . \end{aligned}$$

Thus, we obtain time-dependent speckle pattern distribution ${E (x,y,\Delta ,t)}$. The overall speckle pattern intensity ${I_{r} (x,y)}$ that is evaluated by integrating ${E_{r} (x,y,\Delta ,t)}$ over the pulse duration *T* is given by:

5 $$\begin{aligned} I(x,y)=\int _T{{\left| E(x,y,z,t) \right| ^{2}}}dt=\int \limits _{-\infty }^{\infty }{{\left| S\left( 2\pi \nu \right) E(x,y,z,2\pi \nu /c) \right| ^{2}}}d\nu . \end{aligned}$$

Depending on the shape of the time-domain spectrum the resulting speckle image can change considerably. Below we present a comparison of speckle images obtained using monochromatic and pulsed THz radiation. We confine our modelling by using two types of pulses. First type has Gaussian spectrum shape with central frequency $\nu _0=0.7$ THz and can be described as (the blue curve in Fig. 1g):

6 $$\begin{aligned} S_{1}(\nu )=2\tau _{p}\sqrt{\pi }\exp \left( -4\pi ^2(\nu -\nu _{0})^2\tau _{p}^2\right) , \end{aligned}$$

where $4\tau _{p}\sqrt{\log {2}}$ is the duration of the pulse, ${\nu _{0}}$ is the central frequency of the pulse. The second type of pulse has asymmetric spectrum shape (an asymmetric spectrum is usually observed in experiments) with central frequency ${\nu _{0}}=0.5$ THz (the red curve in Fig. 1g):

7 $$\begin{aligned} S_{2}(\nu )=10^{10}\left[ \sin (0.1\nu ) \right] ^{6}\exp ^{-12\nu }. \end{aligned}$$

Some typical speckle patterns for monochromatic and pulsed THz radiation are shown in Fig. 1d–f. The frequency of the monochromatic THz radiation was taken as $\nu =0.7$ THz, the duration of THz Gaussian pulse was 2 ps. For the plate with random rough surface on its back side with refraction index ${n}=1.55$ for THz spectral range was used, and parameters of the rough surface were chosen as ${\hbox{h}_{{RMS}}}=0.3$ mm and ${Cl}=0.9$ mm. The distance between random phase screen and speckle pattern observation plane was $\Delta =5$ cm.

As it can be seen from Fig. 1, speckle patterns significantly vary depending on type of radiation [monochromatic (Fig. 1d) and pulsed (Fig. 1e,f)] and shape of pulse spectrum (Fig. 1g). Comparing the speckle images in Fig. 1d–f it is noticeable that the number of speckles in Fig. 1e,f which is produced by THz pulses with spectrum ${\hbox{S}}_{1}$, has been increased dramatically. It can be explained by the fact that different spectral components of the pulse produce different speckle patterns which overlap each other. This increases the overall number of speckles in the image which is the advantage of the pulsed radiation over the monochromatic one. However, the shift of the central frequency of the pulse from ${\nu _{0}}=0.7$ THz in ${S}_{1}$ to ${\nu _{0}}=0.5$ THz in ${S}_{2}$ leads to the decrease of contrast of speckle image, forming a lighter background comparing to speckle images for monochromatic THz radiation and THz pulses with spectrum ${S}_{1}$. Shift of the central frequency of the pulse from ${\nu _{0}}=0.7$ THz in ${S}_{1}$ to ${\nu _{0}}=0.5$ THz in ${S}_{2}$ does not make significant changes because of using the same total spectrum range (Fig. 1g). Broadband radiation forms a lighter background comparing to speckle images for monochromatic THz radiation and THz pulses with spectrum ${S}_{1}$.

### Statistical characteristics of speckle patterns

Due to the extensive use of speckle patterns in the optical range^1–8^, such possibilities of application of speckle patterns in the THz frequency range is needed to be considered. For the qualitative applications of speckle structures in the THz range, it is necessary to analyze their characteristics. In this section we describe some characteristics of speckles formed by the propagation of broadband THz radiation through the random phase screen.

**Figure 2:**
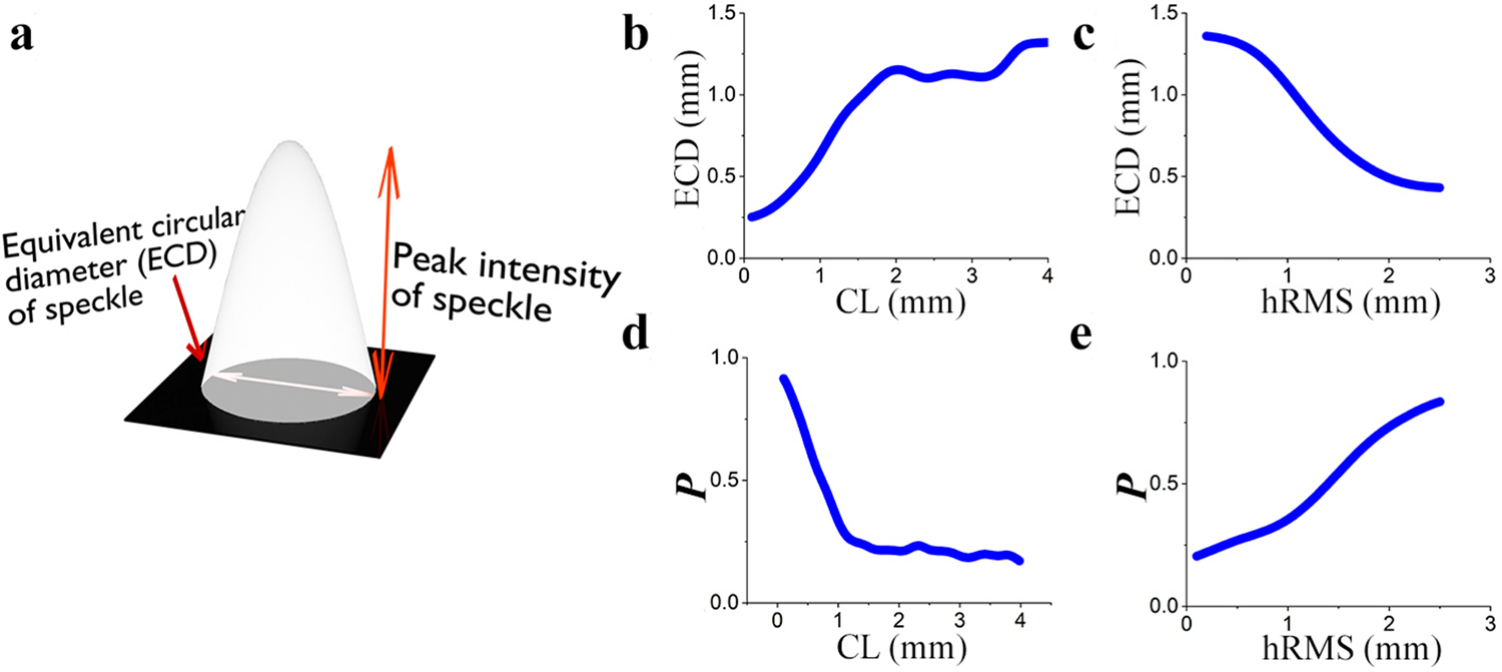
A graphical representation of speckle pattern unit cell of transverse intensity distribution and its parameters. All dependences are plotted for the Gaussian field of broadband THz radiation with a central frequency of radiation 0.7 THz (6) (**a**); dependence of equivalent circular diameter of speckle on correlation length of surface (**b**), dependence of equivalent circular diameter of speckle on RMS of surface height (**c**); dependence of sharpness of speckles (P (8)) on correlation length of surface (**d**), dependence of sharpness of speckles (P (8)) on RMS of surface height (**e**)

For statistical characteristics of speckle patterns generated by THz pulsed radiation, we considered the two parameters. The first parameter is an equivalent circular diameter (*ECD*) of a single speckle. This parameter is usually responsible for the spatial resolution of the reconstructed picture by various methods using speckles, for example^28^. The second parameter is described by the following equation:

8 $$\begin{aligned} P=\left\langle \frac{I_{peak}}{ECD} \right\rangle , \end{aligned}$$

where $I_{peak}$ is a peak intensity of a speckle, $\langle \cdot \rangle$ denotes an ensemble average over *m* single speckles. Parameter *P* can be considered as an average degree of sharpness of the speckle pattern. This parameter is more responsible for the contrast of the speckle patterns. The quantity *P* should increase in order to obtain single speckles that are more distinct in intensity (amplitude). The dependence of these parameters on root mean square deviation of the surface height ${h_{RMS}}$ and correlation length *Cl* is presented in Fig. 2.

As it can be seen from Fig. 2b, the increase of correlation length leads to the magnification of the speckle size^41^, wherein the saturation occurs at the certain value of the correlation length, after which the speckle size remains approximately constant. It can be explained by the fact that by considering broadband pulsed radiation, the use of a field with certain wavelengths affects the speckle size. In this case, the speckle size becomes comparable with the maximum wavelength containing in the spectrum of THz pulses. Moreover, according to Fig. 2c, increasing the root mean square deviation of the surface height leads to a decrease of the speckle size. An increase in the surface roughness after a certain value leads to a decrease of the speckle size, when the phase difference of the field at the minimum and maximum heights of surface described by Eq. (3) becomes greater than 2$\pi$. This was observed in the work^41^. For speckles formed by broadband radiation, the contribution of adjacent frequencies takes place. After passing the element of inhomogeneity the steepness of the front increases so much that a strong change in the phase difference arises at neighboring points, which leads to the decrease of the speckle size. Thus, a strong increase in surface roughness (phase greater than 2$\pi$) is equivalent to the decrease in the correlation length. As one could expect, the sharpness of the speckles is decreasing with the growth of the correlation length as it is shown in Fig. 2d. At the same time the value of speckle sharpness grows with magnification of the root mean square deviation of the surface height due to the strong phase shift for the whole spectrum, see Fig. 2e. The study of these parameters makes an important contribution to the possibility of using formed speckle patterns in various applications. It is essential that the speckle diameter (*ECD*) is responsible for the spatial resolution of the images, and P is responsible for the image contrast. Thus, it is possible to control changes in the formed speckles by changing various characteristics in the phase object.

## Experimental verification

**Figure 3:**
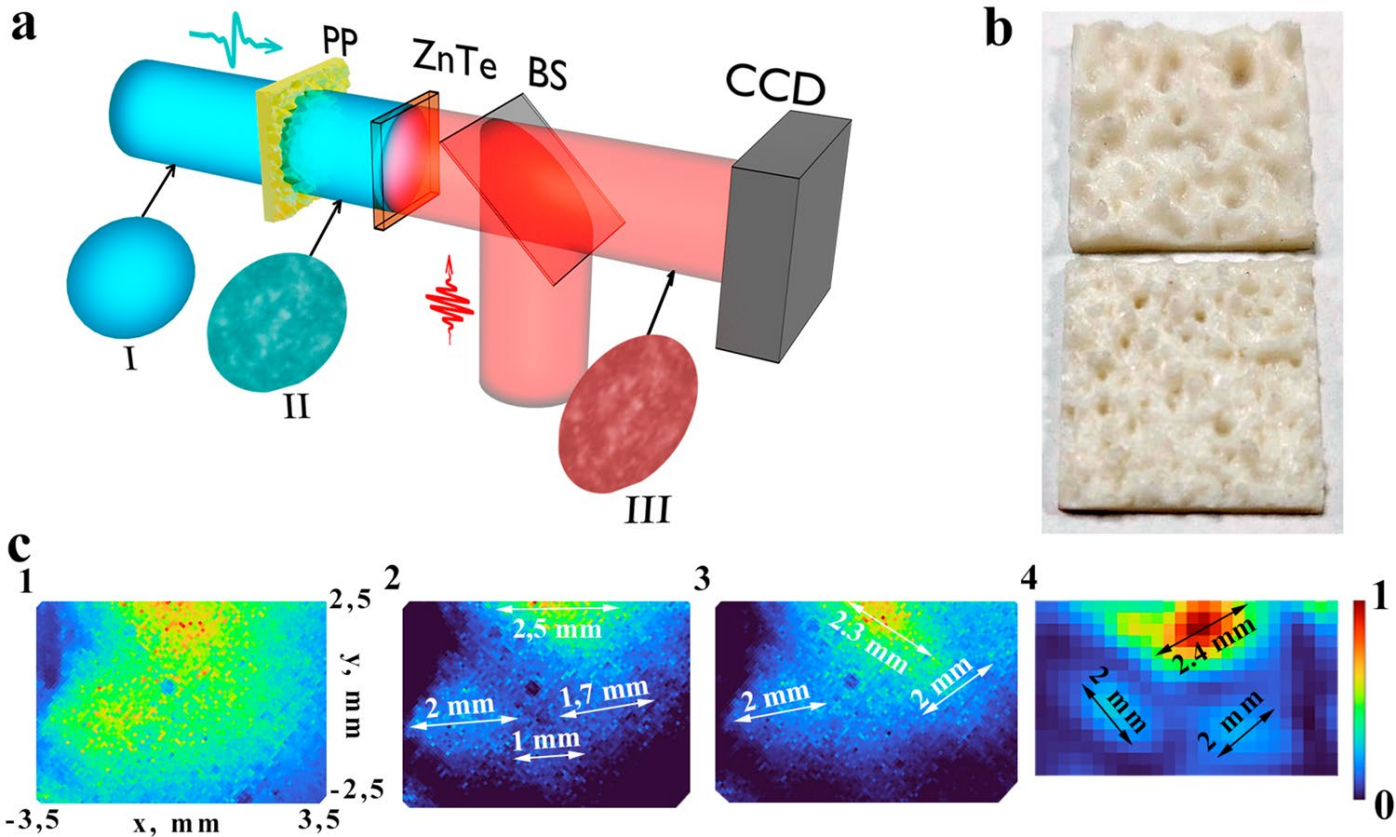
(**a**) Schematic diagram of detection system in THz imaging setup. The Gaussian THz field (I) passes through the transparent phase plate with a random distribution, where it changes its spatial distribution (II). After that, the THz field is detected by the EOS in each pixel of the camera using probe beam (III). In this experiment, the probe beam changes its time delay relative to the THz field and makes it possible to detect the time profile of the THz pulse. (**b**) Photo of the phase plates made from ABS plastic (n(THz) = 1.55) [parameters: $h_{RMS}=0.8\,$mm and ${Cl}=1.5$ mm (top), $h_{RMS}=0.6$ mm and ${Cl}=0.9$ mm (bottom)]. (**c**) Initial THz profile (**1**) and resulting THz speckle patterns obtained from the radiation intensity recorded on an imaging setup for the case in which THz radiation propagates through the phase plates (**2** is **b** (top) and **3** is **b** (bottom)) located at a distance of 5 cm from the ZnTe crystal, **4**—typical result of numerical calculation of speckle pattern formation for the same conditions as in **c3**

THz imaging setup was used for experimental verification of broadband THz field speckle patterns formation. Schematic diagram of detection system is shown in Fig. 3a (A detailed description of the experimental setup is presented in the work^43^). The terahertz radiation was generated due to optical rectification of femtosecond pulses in lithium niobate crystal^44^. The femtosecond pulses from a regenerative Ti-sapphire amplifier (pulse duration 35 fs, energy 1.1 mJ, 1 kHz repetition rate, 800 nm central wavelength) were used as pump radiation. The pulse energy was divided into two parts using a beam splitter (1:49 for probe and pump beam respectively). The probe pulse passed through the delay line and was detected by an electro-optical system (EOS). In this case, a $18\times 18\times 1$ mm ZnTe crystal was used.

The generated THz field with the parameters given below (see “Methods” section) had a Gaussian profile [Fig. 3c(1), a(I)] and passed through the phase diffused plates which were printed from the plastic with the following parameters: a refractive index in the THz range ${\hbox{n}}_{{THz}}=1,55$ (photos of phase objects are shown in Fig. 3b), ${h_{RMS}}=0.8$ mm and ${Cl}=1.5$ mm (shown at the top of Fig. 3b), ${h_{RMS}}=0.6$ mm and ${Cl}=0.9$ mm (shown at the bottom of Fig. 3b). One of the key requirements for the material from which the diffusers are made of by 3D printing to form speckle patterns with THz radiation is its transparency and a sufficiently high refractive index. In principle, other polymeric materials can also be used for these purposes, for example Tsurupica^45^, polylactide (PLA)^46^, Teflon^47^. The parameters of the phase object were selected in relation to the studies carried out in the previous section (*Statistical characteristics of speckle patterns*) for later comparison of the size of the speckles with the numerically calculated ones. Then, the modulated THz field (shown on the II Fig. 3a) was detected by ZnTe crystal, where the real valued spatial distribution of THz pulse in temporal domain^48^ was measured. After that EOS allowed to completely detect the entire picture of THz radiation by using a probe femtosecond field (shown on the III Fig. 3a) and register it on the CMOS camera (CCD). Processing of the experimental results included: averaging in each pixel of the frame by 10 scans to increase the signal to noise ratio. Then Fourier transform was done for each pixel in order to obtain spectra at each point of the frame. Finally the radiation energy in each pixel was calculated by multiplying the amplitude at the corresponding frequency.

As it can be seen from Fig. 3c(1), a THz field with a slightly shifted Gaussian profile was suppressed at the input. However, after passing through the plates, a changes in the spatial distribution of the THz radiation intensity can be noticed . Local maxima are clearly observed, which can be attributed to individual parts of the speckle structure. Moreover, the sizes of these peaks are within the segment of 1–2 mm (Fig. 3c2,c3) which corresponds to the estimates given in Fig. 2b,c. For example, typical numerical calculation result for the parameters selected in Fig. 3c3 is shown in (Fig. 3c4). It can be seen from the figure that typical speckle sizes are also in the range of 1–2 mm. Both results (numerical calculation and experimental one) correspond to the 1–2 mm segment of speckle size calculated on the basis of the equations given in works^49,50^. The experimental data plots are similar and have a correlation coefficient of 0.9. Due to the inhomogeneous Gaussian profile of the probe optical beam that affects the speckle patterns in the THz range during registration, sizes of individual speckles (1–2 mm) and recorded field (5 mm) the high correlation coefficient is obtained. Thus, the possibility of speckle formation by broadband THz radiation has been experimentally confirmed in the work.

## Application in ghost imaging algorithm

In ghost imaging technique classical laser beams typically are used to reconstruct ghost images. In this work we employ speckle patterns obtained numerically for both monochromatic and broadband pulsed THz radiation (“Mathematical model” section). The scheme of ghost imaging utilized in our model is a classical pseudo thermal light ghost imaging setup shown in Fig. 6 (see “Methods” section). In this scheme THz radiation is incident onto the rotating plate with random rough surface on its back side, thus representing a random phase screen, and it forms speckle-picture.

In this paper the ghost image reconstruction is considered for both monochromatic and broadband pulsed THz radiation. For the monochromatic case we use frequency-dependent speckle patterns $I_r(x,y,\nu )=\left| E_{r}(x_{2},y_{2},z=\Delta ) \right| ^{2}$ from the Eq. (11), and for the case of the pulsed THz radiation we use speckle patterns described by Eq. (5) by taking into account all spectral components of the pulse. For ghost image reconstruction we calculate cross-correlation function^51^ between the speckle pattern $I_{r}$ and the intensity transmitted through the object $B_{r}$, which is measured by a point detector and evaluated by means of the following equation:

9 $$\begin{aligned} B_{r}=\int dxdyI_{r}(x,y,z=\Delta )T(x,y), \end{aligned}$$

where *T*(*x*, *y*) is the transmission function of the object. Using a large number of realizations of speckle structures, one can calculate the image estimation function *G*(*x*, *y*) in the following form:

10 $$\begin{aligned} G(x,y)=\frac{1}{M}\sum (B_{r}-\left\langle B \right\rangle )I_{r}(x,y)=\left\langle B\cdot I_{r}(x,y) \right\rangle -\left\langle B \right\rangle \left\langle I_{r}(x,y) \right\rangle . \end{aligned}$$

Here $\left\langle \cdot \right\rangle \equiv 1/M\sum _{r}$ deotes an ensemble average over *M* realizations of ghost imaging algorithm.

**Figure 4:**
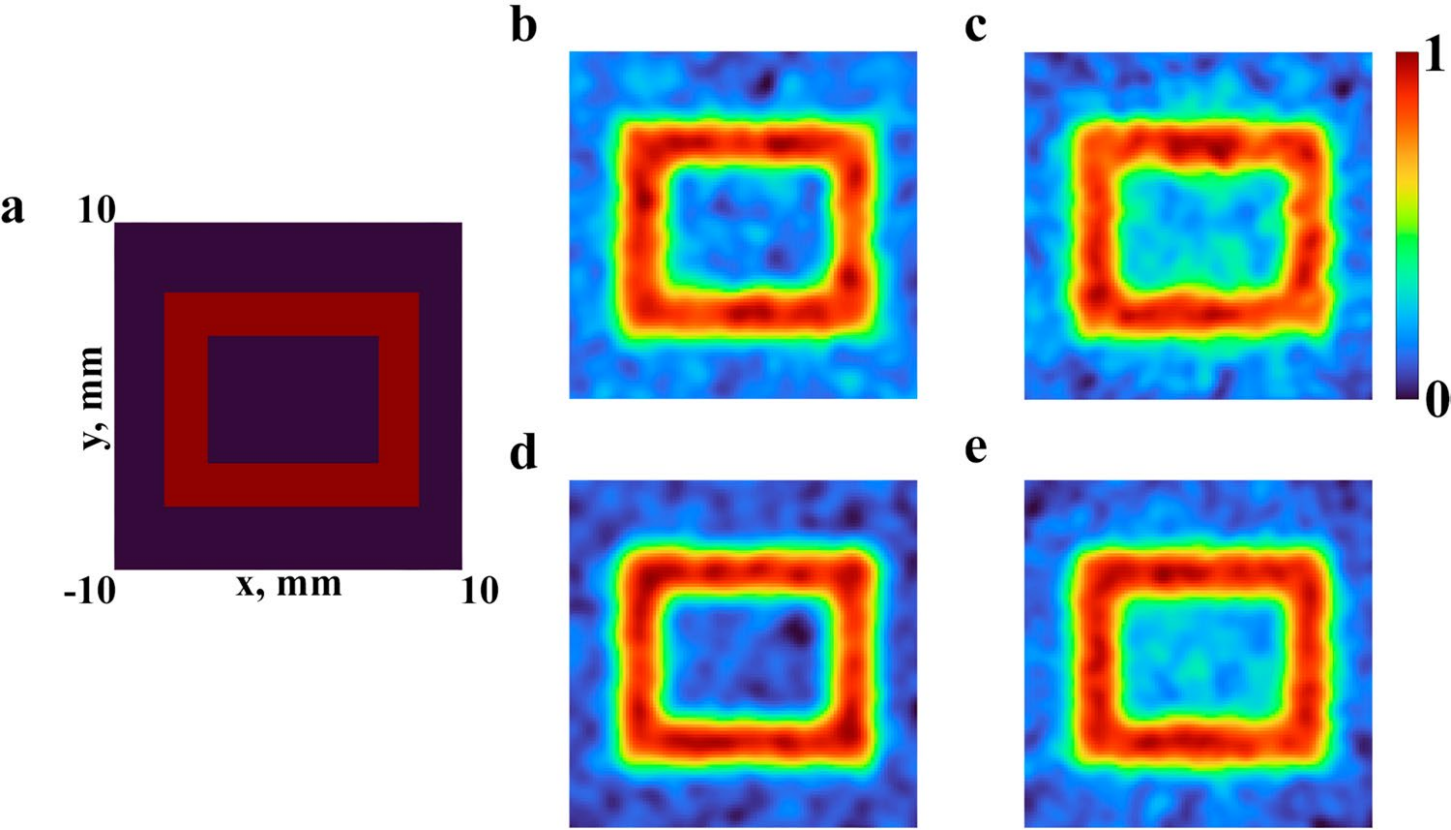
Results of a numerical experiment for ghost imaging. Reference object (**a**), reconstructed images from data with a monochromatic radiation (**b**,**d**) and wide spectrum (**c**,**e**) for 10 (**b**,**c**) and 20 (**d**,**e**) thousand realizations

As an object to be imaged it was used a rectangular ring, shown in Fig. 4a, defined by the inequalities $a_x<|x|<b_x$, and $a_y<|y|<b_y$, in which the geometric parameters are determined by the following values $a_{x}=4$ mm, $b_{x}=6$ mm, $a_{y}=3$ mm, $b_{y}=5$ mm, respectively. Numerical results of ghost image reconstruction for the monochromatic THz radiation are obtained for frequency $\nu =0.7$ THz and shown in Fig. 4b,d for different number of realizations of the ghost imaging algorithm, $M=10{,}000$ and $M=20{,}000$, respectively. Parameters of the random phase screen were chosen as follows: ${h_{RMS}}=1.0$ mm, ${Cl}=1.0$ mm, ${n}=1.55$. The choice of parameters was carried out on the basis of the estimates made in previous section (*Statistical characteristics of speckle patterns*). It was necessary to obtain such a ratio of parameters at which the speckle diameter (*ECD*) was close to the minimum and the value of parameter *P* was high. The distance between the random phase screen and the registration plane is $\Delta =5$ cm. The signal-to-noise ratio is evaluated in the same manner as it was suggested in^51^. The ghost image reconstruction results for the pulsed THz radiation for the same parameters of the random phase screen were obtained for the Gaussian pulse with duration of 2 ps and spectral profile with central frequency $\nu _{0}=0.7$ THz as shown with blue curve in Fig. 1g. Reconstructed images for different number of realizations of ghost imaging algorithm, $M=10{,}000$ and $M=20{,}000$, respectively, are presented in Fig. 4c,e.

Figure 4c,e demonstrates the fact that speckle patterns formed by broadband THz radiation can be used for ghost imaging technique. Now we can see clearly that the increase of the number of realizations of ghost imaging algorithm in Fig. 4, as one could expect, leads to a growth of the quality of reconstructed image that is also in accordance with rise of SNR^8^. In case of image reconstruction with pulsed THz radiation the values of SNR of obtained images are about 10% lower comparing with the corresponding frequency domain results. When comparing monochromatic and pulsed radiation, the same contrast ratio is observed for other central radiation frequencies. It can be explained by the fact that speckle patterns from different spectral components overlap each other. This leads to a decrease of the sharpness of the reconstructed images.

The results presented in this paragraph were made for a speckle pattern correlation coefficient below 0.01. In the experiment we have demonstrated that the correlation coefficient between the speckle patterns is equal to 0.9. For this purpose, in the methods, we showed a comparison of using speckle patterns with correlation coefficient below 0.01 and equal to 0.9. As it was shown by the simulation results, the image reconstruction by the ghost imaging method is also possible even if the correlation coefficient is 0.9. However, in this case, as the correlation coefficient increases, the image quality is getting lower.

## Conclusion

In this study, the possibility of experimental formation of speckle patterns using pulsed broadband THz radiation was demonstrated. Numerical modeling was used to describe the conditions for the formation of a speckle structure with the help of a transparent phase object. It was shown that changes in the statistical characteristics of speckles (sizes and contrast of speckles) are significantly determined by the spectrum of the pulsed THz field and the characteristics of the phase object. The characteristics of the phase object include the horizontal correlation length of surface and the root-mean-square deviation of the surface height. We guess that the minimum speckle size is still limited by the maximum frequency in the THz field spectrum. An increase in the correlation length leads to an increase of the speckle size, while saturation occurs at a certain value of the correlation length, after which the speckle size remains approximately constant. Moreover, an increase in the root-mean-square deviation of the surface height leads to the decrease of the speckle size. At the same time, the value of speckle sharpness increases with an increase of the root-mean-square deviation of the surface height due to a strong phase shift for the entire spectrum. Using theoretical estimates, an experimental verification of the formation of speckle patterns for a pulsed THz radiation was carried out. According to the statistical characteristics, the experimentally obtained speckle patterns correspond to the numerically calculated results. Experimental verification demonstrates, in principle, the possibility of forming speckle patterns using broadband THz radiation. Moreover, the work has numerically demonstrated the opportunity of using such speckles in the image restoration system using the ghost imaging method. It must be said that the use of broadband radiation reduced the contrast of the restored images by only 10%. But at the same time, a broadband radiation will allow in the future to restore not only the image of an object itself, it can also control its spectral properties. This feature is very widely used in various applications of science and technology.

## Methods

### Methods of THz radiation beam propagation

The famous Fresnel propagation equation is described by:

11 $$\begin{aligned} E(x_{2},y_{2},z=\Delta )=\frac{\exp (ik\Delta )}{i\lambda \Delta }\int \int {E(x_{1},y_{1},z=0)\exp \left( i\frac{k}{2\Delta }\left[ (x_{2}-x_{1})^{2}+(y_{2}-y_{1})^{2} \right] \right) }dx_{1}dy_{1} \end{aligned}$$

where ${x_{1},y_{1}}$ are the source plane coordinates, ${x_{2},y_{2}}$ are the observation plane coordinates, $\Delta$ is the distance between source and observation plane (distance of the beam propagation). Here $E_{r} (x_{1},y_{1})$ and E$_{r}$ $(x_{2},y_{2})$ is the field distribution in the source and observation plane, respectively. The integral [main text Eq. (3)] was evaluated numerically by means of FFT algorithm.

In the second method we utilized angular spectrum approach (plane wave decomposition^1^).

12 $$\begin{aligned} E(x_{2},y_{2},z=\Delta )=F^{-1}\left[ H(\nu _{1},\Delta ) \cdot F\left[ E(x_{1},y_{1},z=0) \right] \right] \end{aligned}$$

where $\nu _{1}=(\nu _{x_1},\nu _{y_{1}})$ is a spatial frequency of source plane, *F* and $F^{-1}$ are direct and inverse two dimensional Fourier transforms, respectively, *H* is the transfer function of free-space propagation $H(\nu _{1},\Delta )=\exp (ik\Delta )\exp (-i\pi \lambda \Delta (\nu _{x_{1}}^{2}+\nu _{y_{1}}^{2}))$.

### Experimental parameters of THz radiation

**Figure 5:**
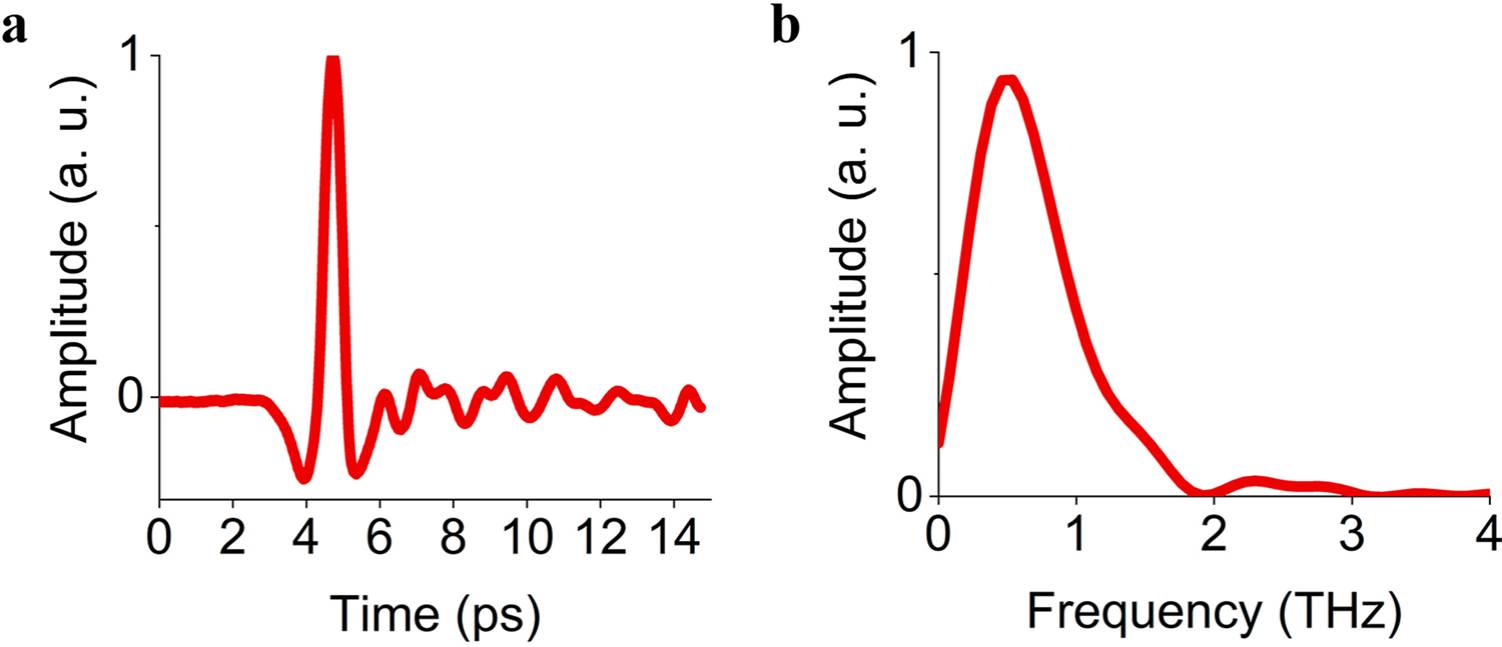
(**a**) The timeform of experimental THz pulse. (**b**) Spectrum of experimental THz radiation

The timeform of THz pulse and its spectrum are shown in Fig. 5. The duration of a THz pulse is 1 ps, and the spectrum range is from 0.05 to 2 THz, pulse energy 300 nJ, the spatial dimension of the Gaussian profile at half-height (FWHM) is 5 mm (Shown Fig. 3c1).

### Model of ghost imaging for pulsed THz radiation

**Figure 6:**
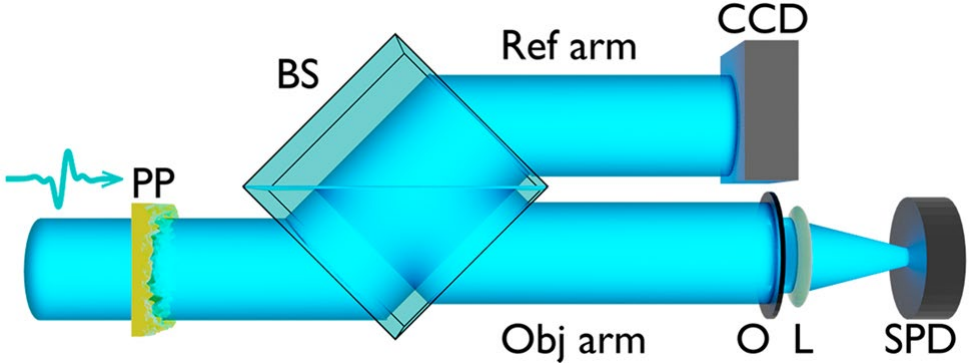
Model of ghost imaging for pulsed THz radiation. THz pulses are incident onto the plate with changing random rough surface (PP) on its back side, representing a random phase screen. The random phase screen introduces a phase shift which subsequently leads to the appearance of random speckle patterns. After that beam is divided into two parts by beam splitter (BS), the first beam interacts with the object (O) and propagates to the single pixel detector (SPD). The second beam is recorded by CCD camera without interacting with the object

The scheme of ghost imaging has a classical pseudo thermal light ghost imaging setup. Here THz pulses are incident onto the plate with changing random rough surface on its back side, representing a random phase screen. The random phase screen introduces a phase shift which subsequently leads to the appearance of random intensity distributions (speckle patterns). After that the beam is divided into two parts by beam splitter. The first beam interacts with the object and propagates to the single pixel detector. The second beam is recorded by multi pixel detector without interacting with the object. Then the cross correlation function between speckle patterns registered by multi pixel detector and overall intensity of speckle patterns transmitted through the object is calculated^51^ (Fig. 6).

Formation of a large number of speckle patterns can be organized using one or two rotating discs, as in work^28^. At the same time, in contrast to this article, which used monochromatic THz radiation and a metal disk creating amplitude modulation of the transmitted radiation, our study demonstrates the possibility of forming speckle patterns using broadband THz radiation by propagating through transparent stochastic phase screens. Another alternative option for arranging spatially inhomogeneous modulation for the formation of speckle structures is the possibility of using the optical range spatial light modulator (SLM) to pump beam modulation which generate THz radiation in crystal^35^ as well as modulation of the probe beam^36^, or fabricating SLM for monochromatic THz radiation based on metamaterials^52,53^. Also, up today, for THz radiation, the use of liquid crystal cells as spatially homogeneous phase shifters is being considered^54–56^.

### Comparing ghost imaging for different correlation coefficients between speckle patterns

**Figure 7:**
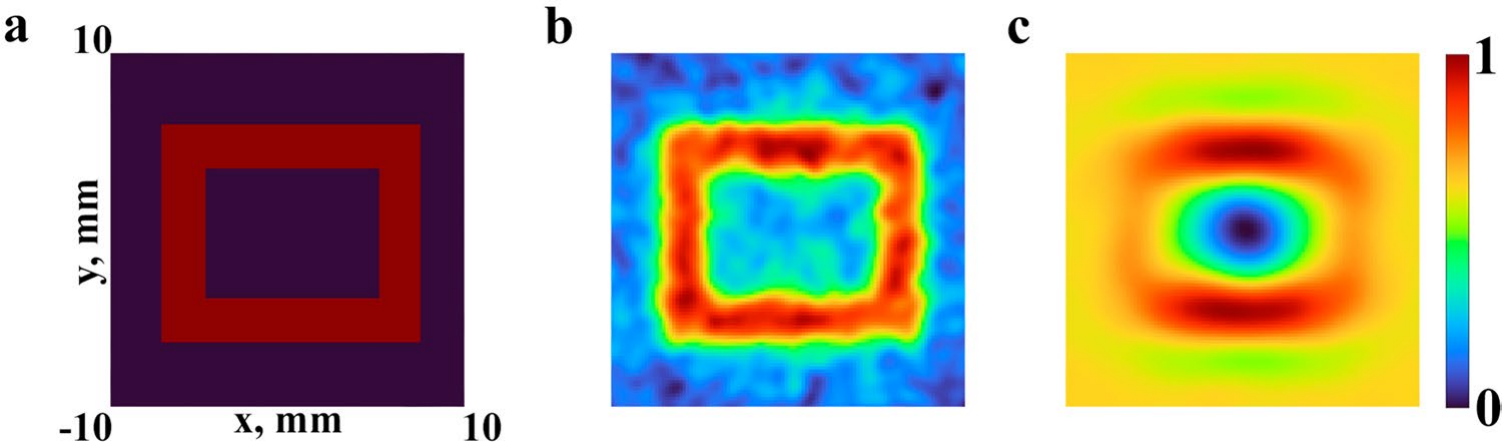
Results of a numerical experiment for ghost imaging. Reference object (**a**), reconstructed images from data with a wide spectrum for 10 thousand realizations using a speckle pattern with correlation coefficients equal to 0.01 (**b**) and 0.9 (**c**)

It is worth noting that the correlation coefficient between experimentally obtained speckle patterns in Fig. 3c2,c3 is approximately equal to 0.9 as they look relatively similar to each other. However, using the numerical model described above, we demonstrate that ghost image reconstruction for the case with the correlation coefficient between two speckle patterns being equal to 0.9 still provides reasonable results. Thus, in Fig. 7 for comparison two reconstructed ghost images are shown with correlation coefficients equal to 0.01 and 0.9, respectively, for the number of realizations N = 10,000. At the same time it should be noted that the quality of the second picture is lower in comparison with the first one.
